# Home Palliative Care for Patients with Advanced Chronic Kidney Disease: Preliminary Results

**DOI:** 10.3390/healthcare3041064

**Published:** 2015-10-28

**Authors:** José L. Teruel, Lourdes Rexach, Victor Burguera, Antonio Gomis, Milagros Fernandez-Lucas, Maite Rivera, Alicia Diaz, Sergio Collazo, Fernando Liaño

**Affiliations:** 1 Nephrology Department, Instituto Ramón y Cajal de Investigación Sanitaria (IRYCIS), Red de Investigación Renal (REDinREN), Hospital Universitario Ramón y Cajal, Carretera de Colmenar km 9100, Madrid 28034, Spain; E-Mails: victor.burguera@salud.madrid.org (V.B.); antonio.gomis@salud.madrid.org (A.G.); milagros.fernandez@salud.madrid.org (M.F.-L.); maiteelizabeth.rivera@salud.madrid.org (M.R.); fernando.liano@salud.madrid.org (F.L.); 2 Hospital Palliative Care Unit, Hospital Universitario Ramón y Cajal, Carretera de Colmenar km 9100, Madrid 28034, Spain; E-Mails: lrexach@segg.es (L.R.); adiaza@salud.madrid.org (A.D.); 3 Home Palliative Care Unit, Hospital Universitario Ramón y Cajal, Carretera de Colmenar km 9100, Madrid 28034, Spain; E-Mail: sergio.collazo@salud.madrid.org

**Keywords:** home palliative care, advanced chronic kidney disease

## Abstract

Healthcare for patients with advanced chronic kidney disease (ACKD) on conservative treatment very often poses healthcare problems that are difficult to solve. At the end of 2011, we began a program based on the care and monitoring of these patients by Primary Care Teams. ACKD patients who opted for conservative treatment were offered the chance to be cared for mainly at home by the Primary Care doctor and nurse, under the coordination of the Palliative Care Unit and the Nephrology Department. During 2012, 2013, and 2014, 76 patients received treatment in this program (mean age: 81 years; mean Charlson age-comorbidity index: 10, and mean glomerular filtration rate: 12.4 mL/min/1.73 m^2^). The median patient follow-up time (until death or until 31 December 2014) was 165 days. During this period, 51% of patients did not have to visit the hospital’s emergency department and 58% did not require hospitalization. Forty-eight of the 76 patients died after a median time of 135 days in the program; 24 (50%) died at home. Our experience indicates that with the support of the Palliative Care Unit and the Nephrology Department, ACKD patients who are not dialysis candidates may be monitored at home by Primary Care Teams.

## 1. Introduction

Kidney disease in which treatment with dialysis has been excluded usually involves complex patients with high comorbidity, often with functional deterioration and occasionally with cognitive deterioration. Healthcare of these patients is characterized by their transfer from one clinic to another of various medical specialties, generally with poor resolution, and by many visits to emergency departments and hospitalizations.

The objective of treatment in this stage of the disease must not be limited to decreasing the rate of renal function deterioration and extending life, but should preferably be focused on achieving the best quality of life possible for the patient and easing the consequences of the disease for the family. These objectives often relegate the conservative treatment of kidney disease to palliative care [1,2].

One aspect that must be kept in mind when providing quality care is the location in which the care will be administered. Repeated visits to healthcare centers are one of the aspects that most negatively affects the patients and their family. Home care may be the most suitable form of care and should be one of our objectives [3,4].

We considered that follow-up of these patients could be performed by Primary Care Teams with the support and advice of the Hospital Palliative Care Unit and the Nephrology Department until more advanced stages of the disease. In December 2011, we implemented a program for advanced chronic kidney disease (ACKD) patient care in which the bulk of the work fell mainly to Primary Care. The program was designed for individuals on conservative treatment and was implemented whenever the patient and their family indicated a preference for home care. In this study, we present our experience from the first three years in which this project has been running and compare it with that from the three years prior to its implantation.

## 2. Material and Methods

Ramón y Cajal Hospital serves a population of 558,000 inhabitants. There are 20 health centers in its catchment area. It has a Hospital Palliative Care Unit and a Home Palliative Care Support Team, each of which contains three doctors and three nurses.

All ACKD patients, whether on conservative treatment or dialysis candidates, are followed with the same treatment protocol in the Nephrology Department’s ACKD clinic. Appointments are scheduled every one to two months. Between 2009 and 2011, 181 ACKD patients on conservative treatment were attended to. The total evolution time (from the first visit until death or until 31 December 2011) was 44,077 days. During this follow-up period, we recorded a total of 332 visits to the emergency department by 117 patients (one visit every 133 days per patient) and a total of 179 hospitalizations of 105 patients (one admission every 246 days per patient). During the period of time analyzed, 64 patients (35%) did not have to visit the hospital’s emergency department and 76 patients (42%) did not require hospitalization. Of the 92 patients who died, 57 (62%) were in the Nephrology Department, three (3%) were in a palliative care hospital, and 32 (35%) were in their home.

In December 2011, the Nephrology Department and the Hospital Palliative Care Unit organized a home care project for ACKD patients on conservative treatment, with the aim of reducing the visits to the Emergency Department and the hospitalizations, and of increasing the number of home deaths.

The program included the following stages:

### 2.1. Patient Detection

We considered all patients with stage 4 or 5 chronic kidney disease who, for different reasons, were not candidates for renal replacement therapy to be eligible for this program. This decision, which required the agreement of the patient and their family, was recorded in the clinical records and in the clinical report issued by the Nephrology Department. At this point, they were offered the opportunity to be included in the home care program, and with their consent were put in contact with the Hospital Palliative Care Unit. Patients and family who did not consent to home care continued to be cared for in the ACKD clinic of the Nephrology Department.

### 2.2. Patient Inclusion in the Home Care Program

The Hospital Palliative Care Unit carried out a comprehensive patient assessment to detect their problems and needs in the different dimensions (physical, emotional, social, cultural, and spiritual), and identified the main care provider.

Functional deterioration was assessed using the Barthel index [5], walking condition was assessed by the Functional Ambulation Category (FAC) scale [6], and the degree of cognitive deterioration by the Global Deterioration Scale (GDS) [7]. If the Barthel index score was less than 60, it was considered that the patient was dependent for basic activities of daily life (from 40 to 55 the degree of dependency was considered to be moderate, from 20 to 35 dependency was severe, and with less than 20 dependency was total). The FAC walking scale has six levels (0: incapable of walking; 1: walks with difficulty holding onto another person; 2: walks with the support of another person; 3: can only walk under supervision; 4: walks independently on a flat surface but cannot climb stairs; 5: walks independently on a flat surface and can climb stairs). The GDS has seven stages (stage 1: without cognitive alteration; stages 2 and 3 indicate mild cognitive deterioration; stage 4 indicates moderate cognitive deterioration; stage 5 corresponds to moderate-severe cognitive deterioration, requiring care in a short period of time; and stages 6 and 7 represent severe or very severe levels of cognitive deterioration).

In addition to the cognitive status, functional and walking capacity of the patients, the study also takes into account the difficulty they face to get to the healthcare centers and the associated problems, for both patients and relatives, these trips may cause. If the patient was suitable for the home care program, a care plan was drawn up and the patient was included in the program records. Subsequently, their Primary Care doctor was contacted by telephone to inform them of patient inclusion, the actions were carried out, and the care that the patient required from then onwards was provided through the program. The Nephrology Department reports were sent by fax, the comprehensive assessment was carried out by the Hospital Palliative Care Unit, and a line of communication was established by telephone and e-mail with the doctors responsible for the program in both services.

Also, the Home Palliative Care Support Team received full information of all the patients included in the program, but its intervention was always determined by a decision made by the Primary Care Team. If the Primary Care doctor had problems with the management of a patient at home, he would ask the Home Palliative Care Support Team for help.

After reviewing all the aforementioned programs with various specialists, we reduced them to those that were absolutely necessary.

### 2.3. Patient Follow-up

The patients were mainly cared for by Primary Care professionals, with the support and advice needed from the hospital’s Nephrology Department and the Hospital Palliative Care Unit. The main patient care location was their home, although at the discretion of the Primary Care Team, it could also have been the health center, bearing in mind the patient’s characteristics and ability to travel.

The Hospital Palliative Care Unit was in charge of coordinating the other teams and following up with the patients by monthly telephone contact with them or their care provider and professionals.

At least one blood test was carried out every three months with the following parameters: complete blood count, creatinine, urea, glucose, sodium, potassium, calcium, phosphorus and ferritin. The glomerular filtration rate was estimated using the formula MDRD-4 IDMS. Blood was taken for scheduled blood tests mainly at the patient’s home. The results were received by the Hospital Palliative Care Unit via the hospital information program and sent to the nephrologist, who made the appropriate treatment changes and provided the patients with the prescriptions dispensed by the hospital (mainly erythropoietin).

All the patients were treated at the Nephrology Department’s ACKD clinic whenever the Primary Care Team, the Hospital Palliative Care Unit, or the patients considered it necessary due to presentation of the main complications related with renal insufficiency such as anemia, hyperkalemia and cardiac insufficiency.

In order to facilitate flexible contact, the patient and their family were provided with the telephone numbers of the Hospital Palliative Care Unit and the ACKD clinic. As indicated previously, the Primary Care professionals were also given these telephone numbers and the e-mail addresses of the nephrologists responsible for this program to enable them to make any consultations they deemed relevant.

### 2.4. Hospitalization

If the patient, in the opinion of the Primary Care doctors or the Hospital Palliative Care Unit, needed to be hospitalized (mainly for uncontrolled cardiac insufficiency and toxic hyperkalemia), we tried to facilitate direct access to the Nephrology Department, thus avoiding as much as possible having to resort to the Emergency Department. Hospitalized patients were monitored in coordination with the Hospital Palliative Care Unit.

### 2.5. Referral to a Palliative Care Hospital

To refer a patient to a palliative care hospital, a previous agreement between the Primary Care Team, Home Palliative Support Team, and Hospital Palliative Care Unit was required, and this last unit was in charge of the procedural management. There were two scenarios in which this protocol was necessary: first, when the patient care needs chronically exceeded the capacity of the home care (acute episodes were treated at Ramon y Cajal Hospital), and second, when the main care provider and the rest of the relatives were unable to provide sufficient care.

### 2.6. Summary of the Functions of Each Team

Nephrology Department:

- To select the appropriate candidates.
- To establish the form of contact (e-mail or telephone) with the Primary Care Team and the Hospital Palliative Care Unit to follow the patients.
- To supervise the periodic analytical results and to make any necessary changes in the treatment and prescription of hospital-dispensed drugs (mainly erythropoietin).
- To follow-up with the patients in the ACKD clinic when requested by the Primary Care or Palliative Care doctors.
- To attend to the hospitalized patient in coordination with the Palliative Care Unit.

Hospital Palliative Care Unit:

- To undertake the integral assessment of the patients remitted from the Nephrology Department for the inclusion in the home palliative care program.
- To coordinate with the Primary Care Unit and the Nephrology Department on patient follow-up.
- To make periodic phone calls to patients and Primary Care doctors, and to receive the analytical results.
- To organize admission in a palliative care hospital when necessary.

Primary Care Team:

- The Primary Care professionals are directly responsible for the patients.
- To follow-up with the patients at home with periodic visits and to undertake the analytical controls. Initially a monthly appointment is scheduled. Then the frequency is adapted to the needs of each case.

Home Palliative Support Team:

- To support the Primary Care Team in the management of the more complex patients.

Statistical analysis: The results were expressed as a mean ± standard deviation or as the mean, median, and interquartile range, according to whether or not the variable analyzed had a normal distribution. The Student’s t-test was used to compare normal distributed data. The chi-square test was used to compare non-continuous variables. The survival analysis was carried out using the Kaplan-Meier method.

## 3. Results

Between 1 January 2012 and 31 December 2014, the home care program was offered to 101 ACKD patients on conservative treatment and 76 (75%) agreed to be included in this program and 25 (25%) preferred to continue being attended to in the Nephrology Department.

Of the 76 patients who agreed to be included in the home palliative care program, 48 were males and 28 were females with a mean age of 81 ± 8 years of age (range 55–92). The most common nephropathy was vascular (33%), followed by diabetic nephropathy (28%). Conservative treatment was chosen for kidney disease due to the existence of another pathology that determined a short-term prognosis which was not likely to improve with renal replacement therapy in 61 cases and a significant cognitive deterioration in six patients. In all of these 67 cases, the decision to carry out conservative treatment was agreed to by the patient and their family. The nine remaining patients rejected renal replacement therapy despite it not being formally contraindicated. At the time of inclusion in the program, the glomerular filtration rate was 12.4 ± 5.8 mL/min/1.73 m^2^ (range 4.5–29.5). Stage 4 ACKD was presented by 24% of the patients (18 cases) and 58 (76%) had stage 5 ACKD. Hemoglobin concentration was 10.8 ± 1.7 g/dL and 47 patients (62%) were receiving treatment with erythropoietin-stimulating agents. The Charlson age-comorbidity index was 10 ± 2 (range 6–15).

The 25 ACKD patients who opted to continue being attended to in the Nephrology Department were younger (76 ± 9 years of age), had higher renal function (the glomerular filtration rate was 20.1 ± 6.2 mL/min/1.73 m^2^), and less comorbidity (the Charlson age-comorbidity index was 7 ± 3) (*p* < 0.001 for the three parameters) than the group of 76 patients who opted for inclusion in the home palliative care program. These 25 patients also had a lower grade of dependency (the Barthel index was ≥60 in 20 patients) and a higher grade of independence for walking (level 4–5 on the FAC scale in 21 patients).

The Barthel index, the FAC scale, and the GDS of the 76 patients included in the home palliative program are shown in Table 1.

**Table 1:** Baseline data at the time of inclusion in the program.

**Barthel Index**	
≥60	38 (50%)
40–55	18 (24%)
20–35	10 (13%)
<20	10 (13%)
**FAC Scale**	
Level 0–3	42 (55%)
Level 0	5
Level 1	9
Level 2	10
Level 3	18
Level 4–5	34 (45%)
**GDS**	
Stage 1	39 (51%)
Stage 2–3	22 (29%)
Stage.4–5	9 (12%)
Stage 6–7	6 (8%)

According to the Barthel index, 38 patients (50%) were dependent for the basic activities of daily life (18 with moderate dependency, 10 with severe dependency, and 10 with total dependency).

The walking assessment showed that 34 patients (45%) were independent for walking (levels 4 and 5 of the FAC scale) and that the 42 remaining (55%) required help or supervision to walk (level 0: 5 patients, level 1: 9 patients, level 2: 10 patients and level 3: 18 patients).

Severe or very severe cognitive deterioration was the main reason for advising against renal replacement therapy in six patients (8%). In 39 patients (51%) we did not observe cognitive deterioration, in 22 patients (29%) we observed mild cognitive deterioration, and in nine patients (12%) there was moderate cognitive deterioration.

The total evolution time (from their inclusion in the program until death or until 31 December 2014) was 17,631 days (mean patient follow-up: 232 days, median: 165 days, interquartile range: 50, 329 days). During this follow-up period, we recorded a total of 83 visits to the Emergency Department by 37 patients (one visit every 212 days per patient). The most common cause of referral to the Emergency Department was heart failure (46%). It must be highlighted that 39 patients (51%) did not have to visit the hospital’s Emergency Department during the period of time analyzed (*p* = 0.024 in comparison to the data from the pre-program implementation period). The mean follow-up time of patients who did not have to visit the Emergency Department was 178 days, and the median was 99 days (interquartile range 44, 293 days).

There were 51 hospitalizations of 32 patients (one admission every 346 days per patient), and the most common cause continued to be heart failure (49%). Of the patients, 58% did not require hospitalization (*p* = 0.028 in comparison to the data from the pre-program implementation period); the mean follow-up time for patients in the program who did not require hospitalization was 205 days and the median was 108 days (interquartile range 47, 320 days).

The Primary Care Team’s main point of contact was the Hospital Palliative Care Unit, who they communicated with by regular telephone calls. The Nephrology Department and Primary Care Team regularly communicated via the family of patients who visited the ACKD clinic to submit blood test results and collect the medication adjustment information and the prescriptions of drugs for hospital use. A patient review was requested in the Nephrology Department’s ACKD clinic by the patient or the Primary Care doctor in only five cases. During evolution, the assistance of Home Palliative Care Support was required for 18 patients and nine had to be transferred to a palliative care hospital when home care became impossible in the final stages of the disease or by the express wish of the patient not to die at home. One patient who had initially rejected renal replacement therapy reconsidered his decision and began treatment with hemodialysis. No other patient displayed their intention to leave the home care program and return to receiving specialized care.

**Figure 1:**
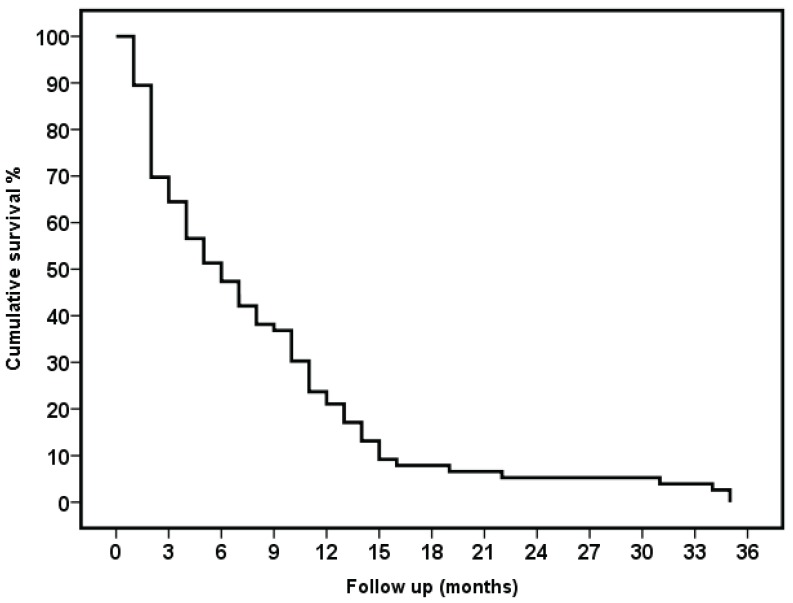
Survival curve.

Figure 1 displays the patient survival curve. Patient death after six months was 45% and after 12 months was 71%. A total of 48 patients died after a mean follow-up time in the program of 179 days, with a median of 135 days (interquartile range 48, 237). In 24 of these patients (50%), the place of death was their home, nine (19%) died in a palliative care hospital, and 15 (31%) in the Nephrology Department of Ramón y Cajal Hospital (*p* < 0.001 in comparison to the data from the pre-program implementation period).

The other 28 patients were still alive on 31 December 2014, with the mean time on the home care program being 322 days, with a median of 233 days (interquartile range 60, 410 days).

## 4. Discussion

Access to renal replacement therapy is not limited in Spain. The coexistence of renal failure with other pathological conditions that lead to short-term mortality or that result in a poor quality of life that cannot be improved with dialysis are the only situations in which the aforementioned treatment is advised against. Even in these cases, the patient’s opinion and that of their family may determine the indication. In this context, patients who accept or choose a conservative treatment of renal failure in its most advanced stage usually have high comorbidity and on many occasions high functional deterioration. The objective of treatment in these cases is to provide the highest level of comfort possible, reducing the impact of the disease on the patient and their family, and home care may contribute to this goal.

Given the low prevalence of ACKD in the general population, the Primary Care Teams are not as used to monitoring these patients as they are with other diseases which, although of the same severity, are more common. Our data show that, with the assistance of the Hospital Palliative Care Unit and the Nephrology Department, both the follow-up of these patients by the Primary Care Teams and mainly home care are possible.

Patients who decided to be included in our home care program were usually elderly (mean age 81 years old), with significant comorbidity (Charlson index with a mean of 10), and had significant walking difficulties (45% required assistance or supervision to walk) and significant functional deterioration (50% were dependent for basic activities of daily life). Travel to healthcare centers is a burden for the patient and their family in this population. However, cognitive deterioration was not a significant problem in these patients, as 80% did not display this problem or it was mild. However, the ACKD patients on conservative treatment who opted to continue being attended to in the Nephrology Department were younger and had better renal function and less comorbidity.

The capacity of Primary Care to assist these patients will depend on the more common healthcare needs at this stage of the evolution of kidney disease. In 2003, Lunney *et al.* reported four functional deterioration models at the end of life: sudden death, functional stability, progressive deterioration, and a fluctuating trajectory [8]. Murtagh *et al.* analyzed the evolution of uremic patients treated conservatively and observed that the most common models were that of functional stability and that of progressive deterioration, and only 21% of patients had a fluctuating trajectory with intermittent relapses [9].

Our experience shows that these patients can be mainly treated by a Primary Care Team over significant periods of time. In the two years of the program, we observed that 51% of patients did not have to visit the Emergency Department and that 58% did not require hospitalization after a median follow-up time of 99 and 108 days, respectively. Regular telephone contact by the Palliative Care Unit with the patient and the Primary Care doctor and communication between the latter and the ACKD clinic allowed the disease to be monitored without the patient having to travel. In 18 patients, it was necessary for the Primary Care doctor to request assistance from the Home Palliative Care Support Team and nine patients required care in an intermediate-term palliative care hospital in the final stage of the disease.

Of the 76 patients included in the care program, 48 died after a median follow-up time of 135 days. After 12 months, 71% of patients had died. The duration of ACKD and the glomerular filtration rate at the time of inclusion in the program were very variable. Due to these aspects and the number of patients, which was low, we did not believe it was appropriate to carry out a factor analysis with a prognosis value. One figure that we should highlight is that almost half the patients (50%) died at home.

When comparing the current results with the previous ones, we have achieved a decrease in visits to the emergency department and in the rate of hospitalizations and an increase in the home deaths with the home palliative care program.

Alonso Babarro *et al.* analyzed the influence of a home palliative care program on hospitalization rates, visits to the emergency department, and the place of death in cancer patients [10]. Our data in ACKD patients on conservative treatment can be favorably compared with those obtained in those with cancer: need for hospitalization (42% *vs.* 66.4%), visits to the emergency department (49% *vs.* 68.1%), and death at home (50% *vs.* 20.8%). The healthcare needs of both pathologies may explain these differences.

Tejedor and Cuevas estimate that the number of candidates for a program of these characteristics varies between 10 and 20 cases/million inhabitants each year [1]. During 2012–2014, 76 patients were assisted, representing an incidence of 45 cases/million inhabitants each year. We must bear in mind that these were the first three years of the program and that we will only know the true incidence in subsequent years.

In all outpatient care and palliative care programs of kidney disease patients, we insist on the need for palliative care training and the previous training of the healthcare workers involved [4,11,12]. Without denying the importance of refresher courses and seminars, continuous contact with Primary Care professionals by the Hospital Palliative Care Team and the Nephrology Department would seem more effective.

Other Spanish authors have developed kidney disease palliative care programs. Leiva-Santos *et al.* propose the extension of the palliative care concept to a complete renal support care program provided by a multidisciplinary team who would act in all chronic kidney disease stages [11]. Our aim was to begin with a modest program that could be carried out with the means available, and whose results were satisfactory in our opinion. The additional effort required for this program was assumed by the teams involved (Primary Care, Palliative Care, and the Nephrology Department), a key aspect for guaranteeing its continuity once the initial enthusiasm had diminished.

This study has some weaknesses and the results should be interpreted with caution. The decision-making was mostly at the discretion of the healthcare providers in the respective components of the program. Neither an analysis of the quality of life of the patients, nor a study of the economic consequences of the implementation of the home palliative care program for patients with ACKD, nor a deep analysis of repercussions of the program on the main care provider and the rest of relatives were carried out. Such input could have added value to the study and should be considered in the design of future studies. However, our work supports the conclusion that palliative care of patients with ACKD is possible if an adequate coordination between different primary, hospital, and palliative care providers is achieved.

The three years of implementation of the palliative program for the management of patients with ACKD, high comorbidity, and/or poor quality of life has taught us several lessons. The first and main one is that a palliative program like this is feasible in daily practice. Others are: (a) the need of a perfect assembly between different pillars (family doctors, palliative care support units, and nephrologists) to carry out the task; (b) that the inclusion of the patients in this program must be preceded with explicative sessions in order to facilitate both its understanding and free acceptance by patients and their relatives. Although not measured in this study, we consider the degree of satisfaction perceived by patients and relatives was high.

## 5. Conclusions

We can conclude that it is possible for Primary Care to carry out home care of uremic patients who are not candidates for dialysis, provided that there is close collaboration with the Palliative Care Team and the Nephrology Department. The fluid communication between all agents involved in the process of caring for these patients and their families is essential.
